# Transition to Kindergarten: Negative Associations between the Emotional Availability in Mother–Child Relationships and Elevated Cortisol Levels in Children with an Immigrant Background

**DOI:** 10.3389/fpsyg.2017.00425

**Published:** 2017-05-02

**Authors:** Constanze Rickmeyer, Judith Lebiger-Vogel, Marianne Leuzinger-Bohleber

**Affiliations:** Sigmund-Freud-InstitutFrankfurt am Main, Germany

**Keywords:** hair cortisol, children with an immigrant background, kindergarten entry, emotional availability, early prevention, mother–child relationship, attachment

## Abstract

**Background:** The transition to child care is a challenging time in a child’s life and leads to elevated levels of cortisol. These elevations may be influenced by the quality of the mother–child relationship. However, remarkably little is known about cortisol production in response to the beginning of child care among children-at-risk such as children with an immigrant background. However, attending kindergarten or any other child day-care institution can for example have a compensating effect on potential language deficits thus improving the educational opportunities of these children.

**Method:** Data of a subsample of *N* = 24 “hard-to-reach” mother–child dyads was collected in the context of the psychoanalytic early prevention project FIRST STEPS. The project focuses on the earliest integration of children with an immigrant background by supporting parenting capacities in the critical phase of migration and early parenthood. Children’s hair cortisol concentration (HCC) was assessed 1 week before (mean age = 38.77 months) and 3 months after kindergarten entry (mean age = 42.26 months). Hair analysis was conducted for both times of measurement, reflecting the first 3 months after kindergarten entry and 3 months prior. Furthermore, the emotional quality of the mother–child relationship was assessed with the help of the Emotional Availability Scales (EAS; Biringen, 2008) shortly before kindergarten entry when the children were about 3 years old (mean age = 37.2).

**Results and Conclusion:** Children’s mean cumulated HCC was higher after kindergarten entry than before. The increase correlated negatively with several dimensions of the EAS. Repeated measures ANCOVA revealed that particularly responsive children and children who had experienced less intrusive mother–child relationships demonstrated lower elevations in HCC after kindergarten entry. Furthermore, a decreased EA score was found in all EA dimensions, besides the dimension “mother’s non-hostility,” indicating problematic EA within the mother–child relationships of the sample. The results suggest that children with an immigrant background who experience more emotional available mother–child relationships seem to regulate stress induced by kindergarten entry more effectively, indicated by lower cortisol elevations after entry. This implicates that supporting early mother–child relationships by intervention may have a positive effect on the children’s ability to regulate stress induced by kindergarten entry thus promoting child development.

## Introduction

The transition from home to child care (e.g., “Kinderkrippe,” “Krabbelstube” or kindergarten)^1^ is an exciting, but stressful time in a child’s life generally leading to increased levels of cortisol (Dettling et al., 1999, 2000; Gunnar and Donzella, 2002; Watamura et al., 2003, 2009; Ahnert et al., 2004). At the beginning of “Kinderkrippe” or kindergarten children are faced with a number of new cognitive, behavioral as well as social challenges. They are separated from their parents or other primary caregivers, must comply with the structure imposed by the new child care setting and have to negotiate relationships with a widening circle of peers.

For recently immigrated families the transition to child care can be a particularly challenging experience (Schaich, 2012; Rickmeyer, 2016; Rickmeyer et al., in press). First of all, feelings of loss and separation can be particularly painful and hardly bearable for these families due to their prior migration (Kogan, 2005; Akhtar, 2007; Leuzinger-Bohleber and Fritzemeyer, 2016; Leuzinger-Bohleber and Lebiger-Vogel, 2016). Secondly, an empathic support of the child during transition and associated separation processes might be complicated by potential burdening life circumstances of these families, difficulties in understanding German as well as potential traumatizing experiences and previous losses (Schaich, 2012; Rickmeyer, 2016; Rickmeyer et al., in press).

Cortisol, the primary glucocorticoid of human and non-human primates alike, is the hormonal product of activation of the hypothalamic–pituitary–adrenal (HPA)-axis – a major part of the neuroendocrine system and physiological stress system. It has been well documented that cortisol production increases in response to stress. Experiences with manageable stress are important for a healthy development. However, the beneficial aspects of stress are diminished when it is so severe that it overwhelms a person’s ability to cope with it effectively (Gunnar and Quevedo, 2007). Particularly early in life overwhelming and enduring challenges imposing on the stress system can have negative and prolonged effects on the brain development and parts of the physical stress system (like the HPA-axis). Research increasingly suggests that severe early life stressors may result in decreased brain volumes, elevated brain connections, dysregulation of the neuroendocrine stress response system, and dysfunction of the limbic system involving regions such as the hippocampus, medial prefrontal cortex, and amygdala (Lupien et al., 2009). This in return can compromise proper functioning of the nervous and immune systems and lead to cognitive deficits and emotional dysregulations in adulthood (Gunnar and Donzella, 2002; Tarullo and Gunnar, 2006; Gunnar and Quevedo, 2007; Gunnar et al., 2009; Lupien et al., 2009; Loman and Gunnar, 2010; Matz et al., 2010; Atzil et al., 2011). In accordance to these assumptions, the dysfunction of the HPA-axis is associated with the development of different diseases as well as mental health problems such as depression (e.g., Kirschbaum, 2001; Heim et al., 2008; Stalder and Kirschbaum, 2012).

Prolonged stress early in life can cause a sensitization of the HPA-axis (Gunnar and Quevedo, 2007), leading to an increased reactivity to stressful events and enduring activation of the physiological stress system and thus to a constantly increased release of cortisol (Charmandari et al., 2003; Lupien et al., 2009). However, several studies demonstrated that children who had experienced severe stress during their first years of life, showed elevated cortisol levels during early childhood but decreased cortisol levels later in life (e.g., Susman, 2006; Tarullo and Gunnar, 2006). Therefore several researchers support the so-called *attenuation hypothesis*. It postulates that in the long run elevations in cortisol due to severe stress early in life lead to a downregulation of the HPA-activity and thus to decreased cortisol levels (Gunnar and Vazquez, 2001; Repetti et al., 2002; Susman, 2006; Trickett et al., 2010).

Normally, the human cortisol follows a circadian rhythm: the cortisol levels are highest at waking, followed by a steady decline across the day, returning to its lowest levels at midnight (Kirschbaum et al., 1999)^2^. Deviations from this pattern are supposed to indicate stress (Kirschbaum and Hellhammer, 1994). A meta-analysis has shown that chronic stress is associated with flatter cortisol slopes across the day, a decreased overall diurnal cortisol level as well as higher cortisol levels in the afternoon and lower levels in the morning (Miller et al., 2007). For example, chronically depressed patients show a decreased or rather flatter decline in cortisol levels across the day (Deuschle et al., 1997; den Hartog et al., 2003). In addition, several studies have demonstrated that lacking positive and supportive relationships with the primary caregivers during childhood can evoke changes of the typical diurnal cortisol pattern, too (Gunnar, 2000; Tarullo and Gunnar, 2006; Roisman et al., 2009). Deviations from the typical diurnal cortisol pattern have been found, for example, in traumatized, neglected and abused children (Deuschle et al., 1997; Gunnar and Vazquez, 2001)^3^. To conclude, previous research indicates how important it is to reduce stress in early childhood and to support children’s ability to regulate stress effectively. Both is important in order to prevent a permanent activation of the HPA-axis as well as a potentially resulting downregulation of this axis.

Research has demonstrated that the time of child care entry as well as the months afterwards are associated with an increased reactivity of the HPA-axis (e.g., Watamura et al., 2002, 2003; Ahnert et al., 2004; Badanes et al., 2012). Thereby increasing cortisol levels from morning to afternoon, in contrast to decreasing cortisol levels across the day for the same children at home, have been found in several studies (Dettling et al., 2000; Watamura et al., 2003, 2009). The rise of cortisol level across the day at child care seems to be stronger for younger children (36–60 months) than for children of school age (older than 60 months; Dettling et al., 1999; Vermeer and van IJzendoorn, 2006; Bernard et al., 2015). Additionally, children seem to need time to adapt to the new situation. For example, Bernard et al. (2015) demonstrated that children even experienced an increase in the cortisol rise at child care across a 10-week transition phase. Although the elevations in cortisol may decrease after several months spent in child care, even 5 months after starting child care children still show higher levels of cortisol at child care compared to spending the day at home (Ahnert et al., 2004).

Previous work has addressed potential short- and long-term negative effects of full-day care (e.g., Belsky et al., 2007; Roisman et al., 2009; Watamura et al., 2010). For example, Watamura et al. (2010) have demonstrated that children who attended full-day child care and who showed increased salivary cortisol levels had lower antibody levels as well as a higher illness frequency. This work suggests that the rising cortisol pattern may have consequences for physical health (Watamura et al., 2010). Additionally, an analysis of NICHD-data^4^ demonstrated that longer child care hours as well as maternal insensitivity during the first 3 years of life are subsequently associated with lower awakening cortisol levels at the age of 15 (Roisman et al., 2009). The authors concluded that this result is in line with the attenuation hypothesis (Susman, 2006) and may indicate long-term negative effects of both low maternal sensitivity and early full-day child care. However, it still remains unclear what (if any) exact consequences alterations of cortisol level due to child care may have. Furthermore, particularly for children growing up in high-risk environments and experiencing less sensitive parent-child relationships positive early child care experiences may provide a buffer against negative developmental outcomes. Thereby, emotional available teachers could serve as secure alternative attachment figures in terms of a resiliency promoting factor (Werner and Smith, 1982, 1992; Shivers, 2008; Hauser et al., 2009).

Research efforts to understand stress reactivity at child care have demonstrated that variables such as the caregiving quality (Tout et al., 1998; Dettling et al., 2000; Sims et al., 2006; Watamura et al., 2009) as well as the attachment to mothers (Ahnert et al., 2004) and teachers (Badanes et al., 2012) influence the increase of cortisol during child care. It has been postulated that through their contingent and sensitive interactions with their primary caregiver, securely attached children develop regulatory capacities that allow them to modulate stress reactions more effectively. Thus they may be better equipped to deal with the stressors associated with child care entry (Nachmias et al., 1996; Loman and Gunnar, 2010). It is assumed that children who experience enough empathy and a good-enough care and satisfaction of needs through their primary caregivers early in life will develop a basic trust (“Urvertrauen” according to Erikson, 1957) as well as adequate affect regulation (Leuzinger-Bohleber, 2009, 2015). This in return helps them to cope with stressors more effectively later in life (Leuzinger-Bohleber, 2015). In the context of the emotional quality of mother–child relationships the construct of Emotional Availability (EA; Mahler et al., 1975) has often been used to describe the capacity of a dyad to share an emotionally healthy relationship (Emde, 1980; Biringen et al., 2014). It can be assessed with the help of the Emotional Availability Scales (EAS, Biringen, 2008). They measure the affect and behavior of both the child and the mother (caregiver) on six dimensions, four focusing on the caregiver (sensitivity, structuring, non-intrusiveness, and non-hostility) and two focusing on the child (child responsiveness to the caregiver and child involvement of the caregiver; Biringen, 2008; Biringen et al., 2014). The term EA has first been used by Mahler et al. (1975) in order to describe a mother’s supportive attitude and presence in the context of infant/toddler explorations away from her. Furthermore, the descriptions and definitions of EA are based on Bowlby’s (1958) attachment theory which posits, that higher levels of parental EA promote more secure infant attachment and responsiveness to the parent (Easterbrooks and Biringen, 2000; Emde, 2000; Lum and Phares, 2005). Significant findings have confirmed the positive relationship between parental EA and children’s attachment security (see review by Biringen et al., 2014). In addition, various studies have demonstrated that samples with a high risk for developmental problems due to various mental health or psychological issues (e.g., mother’s depression, substance abuse, history of maltreatment, etc.) show comparatively lower EA (Biringen et al., 2014). Additionally, negative associations between parental traumatization and a reduced quality of the parent-child relationship have been found (Schechter et al., 2010; Feeley et al., 2011; van Ee et al., 2012).

In the context of EA and stress responses an interesting study by Kertes et al. (2009) looked at preschoolers’ HPA-axis reactivity (evidenced by rises in cortisol in laboratory to social and non-social threat contexts) in response to a threat. They demonstrated that sensitive parenting helped to regulate stress responses of highly inhibited children to social (but not non-social) threat. Another study suggests that even in the context of a routine caregiving task a higher EA is associated with lower stress reactivity and with earlier circadian patterning in very young infants (Philbrook et al., 2014). The authors investigated both EA and cortisol levels (with the help of saliva samples) in infants at the age of 1 and 3 months. The results demonstrated that infants of more emotionally available mothers showed lower levels of cortisol secretion across the night than infants of less emotional available mothers. Another study, conducted by Little and Carter (2005) in the US, presented an emotional challenge to (mostly) African-American mother-infant-dyads with a low socio-economic status (SES) in order to investigate children’s emotion reactivity. Thereby children of dyads with more emotional available mother–child relationships showed greater emotional control and less difficulties in emotion regulation during the challenging situation.

In the context of kindergarten a study by Kang (2005) provides evidence that EA predicts preschool children’s externalizing and internalizing behavior problems at school. The results suggest that children who had experienced less emotional available mother–child relationships appeared to be less well regulated and adjusted than those with higher emotional available mother–child relationships. Furthermore, in a study by Howes and Hong (2008) examining Mexican-heritage families in the US, maternal structuring as well as sensitivity at home when the child was 3 years of age predicted children’s pretend play, social competence as well as children’s exclusion by peers during pre-kindergarten^5^. Additionally, an investigation showed that multiple dimensions of EA measured in the pre-kindergarten year were associated with kindergarten readiness (Biringen et al., 2005). Thereby, the dimensions mother’s sensitivity and structuring as well as child responsiveness and child involvement, assessed before kindergarten entry, predicted teacher-reported internalizing and externalizing symptoms as well as levels of observed aggression and/or victimization during the transition to kindergarten and at the end of the kindergarten school year.

In order to investigate a potential buffering role of attachment security against elevations of cortisol due to child care entry, Ahnert et al. (2004) have assessed attachment security (with the help of Ainsworth’s Strange Situation paradigm; Ainsworth and Wittig, 1969) as well as cortisol levels of infants and toddlers. The assessments took place during the first and last day of an adaptation phase to child care (parent present at child care), during the first, fifth, and ninth day of separation (parent not present) as well as 5 months after child care entry. The dyads were coded for secure-base behaviors and salivary cortisol was assessed in the morning. The children’s mean age at child care entry was 14.9 months. Only when the mother was present during the adaptation phase securely attached young children showed lower cortisol levels across the day at child care compared to insecurely attached children. However, both securely and insecurely attached children showed increased cortisol levels during the separation phase, when the parent was not present, compared to the adaptation phase. In both attachment groups the cortisol levels were lower after 5 months than during the separation phase, but still higher than home baseline levels. These results indicate that a secure maternal attachment has a buffering role against elevations of cortisol at child care, however, only when the parent is present. This is in line with the assumption that it might be of additional importance in coping with potential stressors whether a responsive caregiver is present or not (Bowlby, 1973; Gunnar and Brodersen, 1992). However, it remained unclear whether the same effects would be found in older children (e.g., at kindergarten age) who may have already developed more stable attachment systems. It could be speculated that older securely attached children are already able to cope with impending stressors more effectively on their own than younger securely attached children.

Badanes et al. (2012) investigated the potential buffering role of both attachment to mothers and teachers in *N* = 110 children (age 2.0–5.5 years) at child care, with an inclusive sample of both low socio-economic and Mexican-origin families. In their study children with more secure attachments to teachers were more likely to show falling cortisol or less increasing cortisol levels across the child care day. The attachment to mothers did not have an effect on the cortisol pattern. However, maternal attachment interacted with quality of child care, with buffering effects found for securely attached children attending higher quality child care. Accordingly highly secure attached children with high classroom quality showed the optimal decreasing cortisol pattern across the child care day. However, securely attached children with lower classroom quality showed increasing cortisol levels across the day. Furthermore, insecurely attached children showed a flat pattern across the day regardless of classroom quality. Concerning maternal attachment security the authors concluded that low maternal attachment security may prevent the optimal decreasing cortisol pattern even in high-quality child care environments and that securely attached children at this young age are more distressed by lower-quality child care. In summary, this study suggests that after several months spent in child care the attachment to teachers may play a more important buffering role for potential elevations of cortisol in association with child care than maternal attachment (Badanes et al., 2012).

The effect of the emotional quality of the parent-child relationship on children’s elevations in cortisol after child care entry has not been widely studied, yet. The study by Badanes et al. (2012) included both low socio-economic and Mexican families, but was conducted in the US. Ahnert et al. (2004) investigated younger children from middle-class families in Germany. Thus remarkably little is known about cortisol production in response to the beginning of kindergarten among children with an immigrant background in Germany.

However, starting and adjusting successfully to the challenges imposed by kindergarten^6^ can have a positive effect on the child’s development and future school success (Seyda, 2009) and is therefore particularly important for children with an immigrant background who are still disadvantaged in German educational institutions (Autorengruppe Bildungsberichterstattung, 2016). Apart from this, attending kindergarten can have a compensating effect on potential language deficits thus improving the educational opportunities of these children (Becker and Tremel, 2011). However, as mentioned above, due to previous migration-related losses and parents’ potential traumatizing experiences the transition to kindergarten can be a particularly painful experience for families with an immigrant background (Schaich, 2012). In addition, children from these families are more likely to live in high-risk environments (Moro, 2014). In 2014, 44% of children living in families with an immigrant background grew up exposed to at least one situation of risk such as unemployed, low-income earning or educationally disadvantaged parents (Autorengruppe Bildungsberichterstattung, 2016). Thus it is assumed that these children are more likely to have experienced stressful life events than those children who grow up in families with a higher SES and who have not experienced the unique challenges associated with immigration. A review by Mesman et al. (2012) on observational studies of parental sensitivity in ethnic minority families in the US and the Netherlands with young children indicates that parental sensitivity is generally lower in ethnic minority families than in majority families. However, it suggests that the main cause for this difference is family stress due to socio-economic disadvantages. Apart from stress due to socio-economic factors (low SES, unemployment, insecure institutional status, etc.) challenges due to psychological factors associated with the different phases of migration, particularly mothers’ risk of social withdrawal and isolation, can have additional negative effects on the emotional quality of the parent-child relationship (Lebiger-Vogel et al., 2016; Leuzinger-Bohleber and Lebiger-Vogel, 2016). These potential negative effects in turn can have a negative impact on the children’s ability to regulate stressful events. Thus the risk that children with an immigrant background encounter difficulties coping with stressors associated with kindergarten and show increased levels of stress may be significant. Furthermore, potential separation difficulties in the parent-child relationship due to parents’ migration-related losses could additionally complicate the transition to kindergarten (Rickmeyer, 2016; Rickmeyer et al., in press). However, as mentioned before, remarkably little is known about cortisol production in response to the beginning of German kindergarten among children with an immigrant background. Potential associations between their cortisol responses and their relationships with their primary caregivers have not been investigated, yet. Therefore the purpose of the current study was to assess the role of the emotional quality of the mother–child relationship as a potential buffer against cortisol reactivity after kindergarten entry in a sample of children with an immigrant background.

Most of the previous studies demonstrating associations between maternal attachment security and HPA-axis activation have used Ainsworth’s Strange Situation Test (Ainsworth and Wittig, 1969; e.g., Hertsgaard et al., 1995; Ahnert et al., 2004). However, traditional instruments based on attachment theory such as the Strange Situation Test have been criticized for not taking sufficiently into account cultural variations (Rothbaum et al., 2000; Keller, 2012). Therefore in the current study the emotional quality of the mother–child relationship was assessed with the help of the EAS (Biringen, 2008). The EAS have the advantage that they can be rated independently of the caregivers’ cultural background (for an overview see Biringen et al., 2014; Lebiger-Vogel, 2016) with a more dyadic focus than traditional attachment instruments. There is a large body of empirical research using the EAS. They have been applied in more than 100 studies and a lot of different countries all over the world (Biringen et al., 2014).

In the context of parents’ migration and EA, a previous study by van Ee et al. (2012) demonstrated decreased and problematic EA within mother–child relationships of a sample of *N* = 49 refugees and asylum seekers in the Netherlands. In this study the relationships between maternal posttraumatic stress symptoms, parent-child interaction (using the EAS) as well as infants’ psychosocial functioning and development was investigated. Their results showed a positive relationship between maternal posttraumatic stress symptoms and insensitive, unstructuring or hostile, but not intrusive, parent-child interactions. Furthermore, infants showed lower levels of responsiveness and involvement to their traumatized mothers (van Ee et al., 2012).

In addition, in the current study children’s cortisol levels were assessed with the help of hair cortisol assessment, a new and promising method to measure cortisol (Stalder and Kirschbaum, 2012). Assessments took place approximately 1 week before as well as 3 months after kindergarten entry. HCC assessment offers a measure of cumulative HPA-activity and provides a retrospective reflection of integrated cortisol secretion over periods of several months. In contrast, previous methods to measure cortisol (e.g., saliva, urine, blood collection) are limited in the temporal range of assessment because they provide only snapshots of HPA-activity (Stalder and Kirschbaum, 2012). Only one study published to date in this context has investigated children’s stress level with the help of HCC, yet, however, during the transition to school (Groeneveld et al., 2013)^7^.

The current study was conducted in Frankfurt/Main, Germany, and examined the following questions:

(1) Do children with an immigrant background show higher cumulated levels of cortisol, measured with the help of HCC, during the first 3 months after kindergarten entry than during the 3 months prior?
(2) Is there a link between the emotional quality of the mother–child relationship, measured with the help of the EAS (Biringen, 2008), and a potential increase in cortisol after kindergarten entry?

It has been expected that children over the age of three, who have experienced more emotional available maternal relationships, have already developed better regulated stress systems that help them to regulate stress more effectively. Therefore they should show a smaller increase of cumulated HCC after kindergarten entry compared to children with less emotional available mother–child relationships. Thereby the relationship between a potential increase in cortisol and different EA scales was investigated.

All children and families included in the current study had taken part in the early prevention project “FIRST STEPS – An Integration Project for Infants with an Immigrant Background.” The project was conducted by the Sigmund-Freud-Institut (SFI, Frankfurt/Main) in close collaboration with the Anna-Freud-Institut (AFI, Frankfurt/Main) and the IDeA-Center^8^ (for a detailed project description see Lebiger-Vogel et al., 2013, 2015a,b, 2016; Burkhardt-Mußmann, 2015; Rickmeyer et al., 2015; Leuzinger-Bohleber and Lebiger-Vogel, 2016)^9^. It has first been implemented in Frankfurt/Main and then in Berlin and serves as a scientifically evaluated model project. By using a prospective randomized comparison group design the effectiveness of a psychoanalytically oriented intervention (intervention A) is evaluated in comparison to an intervention being offered by lay helpers (intervention B). Both interventions are offered from the time of pregnancy until the commencement of kindergarten. The psychoanalytic approach is in line with a new form of *outreaching psychoanalysis* (Leuzinger-Bohleber et al., 2011, 2013). This can be viewed as an attempt to transfer psychoanalytic expertise into the field of low-threshold offerings for “children-at-risk” and their families. It has the aim to contribute to early prevention and improving the integration of children deriving from socially marginalized groups of society (Leuzinger-Bohleber, 2014). The mothers who receive intervention A are supported by female psychoanalytically trained project staff^10^. The aim of the intervention is to optimize the earliest mother–child relationship by supporting parenting competences as well as mothers’ integration in order to support the emotional, cognitive, and language development of the children. The mothers of the comparison group take part in self-organized mother–child groups led by lay helpers. The project addresses “hard-to-reach” families^11^, particularly mothers who are either pregnant or recently gave birth (and their husbands) and who have mostly only been living in Germany for a short time and thus have not had the chance to be integrated, yet. In order to evaluate the two interventions a variety of different instruments is applied. Over 300 families have already been recruited in Frankfurt/Main and Berlin. First preliminary EAS baseline data of the FIRST STEPS study (*n* = 45) indicate that the recruited mother–child dyads show a problematic and decreased EA after birth (assessed 2.5–5.5 months after child birth; see Lebiger-Vogel, 2016).

## Materials and Methods

### Participants

The children and mothers included in the current study represent a subsample of the FIRST STEPS study. Included were those children who were old enough at the time of data collection, whose hair samples were long enough and whose mothers had agreed to take part in the HCC assessments as well as video-taping.

All of them had already completed their project participation. Forty-one percent of the children included were female (*n* = 10 of the *N* = 24 children). Children’s mean age approximately 1 week before kindergarten entry was 38.77 months (*SD* = 3.18) and 42.26 months (*SD* = 2.99) at the second HCC assessment (∼3 months after kindergarten entry). Most of the children’s mothers came from Turkey (29.2%), Eastern-European countries (16.7%; Serbia, Bulgaria, and Romania) as well as North-African countries (16.7%; Morocco, and Algeria) and other African countries (16.7%; Ghana, Ethiopia, and Eritrea). Two mothers came from Afghanistan (8.3%), two from Korea (8.3%), and one from Venezuela. Mother’s mean duration of school education was 10.26 (*SD* = 3.39). While 20.8% of the mothers reported no school graduation, 33.3% had completed a higher school education (A-levels/highschool-diploma), and 45.8% had attended secondary school. Their mean duration of school education was similar to the duration of school education of women with an immigrant background who were included in a representative survey of the BAMF (German Federal Office for Migration and Refugees; Rother, 2008). However, the women included in our study had a lower educational status in comparison to the women included in the BAMF-survey. Mother’s mean length of stay in Germany was 7.15 years (*SD* = 3.83). Most of the included mothers had immigrated due to marriage, family reunification, and economic reasons. All mothers were encouraged to remain with their children in the kindergarten settings during a transitional period to help them adapt before the first separation. While *n* = 6 of the children had visited another day care institution (“Krippe”) before kindergarten, *n* = 18 had been cared for by their mothers before kindergarten entry. Most of the children were first (50.0%) or second children (25%; see **Table 1**).

**Table 1:** Sample description of *N* = 24 mothers and children.

**Variables**	***N* = 24**
Children’s age 1 week before kindergarten entry in months (HC1)	Mean = 38.77 (*SD* = 3.18)
Children’s age approximately 3 months after entry in months (HC2)	Mean = 42.26 (*SD* = 2.99)
Children’s age at EAS assessment in months	Mean = 37.20 (*SD* = 2.47)
**Mother’s country of origin**	
• Turkey	29.2% (*n* = 7)
• Eastern-European countries	16.7% (*n* = 4)
• North-African countries	16.7% (*n* = 4)
• Other African countries	16.7% (*n* = 4)
• Afghanistan	8.3% (*n* = 2)
• Korea	8.3% (*n* = 2)
• Venezuela	4.2% (*n* = 1)
**Child rank**	
• First child	50.0% (*n* = 12)
• Second child	25.0% (*n* = 6)
• Third child	12.5% (*n* = 3)
• Fourth child	8.3% (*n* = 2)
• Fifth child	4.2% (*n* = 1)
**Out-of-home child care experience before kindergarten entry**	
• Number auf children without prior out-of-home child care experience	*n* = 18
• Number auf children with prior out-of-home child care experience	*n* = 6
Length of mother’s stay in Germany in years	Mean = 7.15 (*SD* = 3.83)
Mother’s age at child’s kindergarten entry in years	Mean = 32.92 (*SD* = 5.58)
Mother’s duration of school education in years	Mean = 10.26 (*SD* = 3.39)
**Mother’s highest educational level**
(1) A-levels/highschool-diploma	33.3% (*n* = 8)
(2) Secondary education	33.3% (*n* = 8)
(3) Lower secondary education	12.5% (*n* = 3)
(4) No school graduation	20.8% (*n* = 5)

The final study protocol as well as the final version of the written informed consent form were approved by the Ethic Review Commission of the Federal Chamber of Psychotherapists of the State of Hessen, Germany. Written consent was obtained from each participating family and the study has been carried out in keeping with local legal and regulatory requirements (Trial registration in DRKS; ID: DRKS00004632).

### Emotional Availability Scales

The emotional quality of mother–child interaction was assessed on average one and a half months before the first HCC assessment, when the children were approximately 3 years old (mean age = 37.2, *SD* = 2.47). Therefore mother–child interactions were videotaped for 30 minutes either during home visits or in the social institutions where the project’s group offerings take place. During videotaping children were asked to solve a puzzle task with the help of their mothers. All videos were rated by trained psychologists with the help of the EAS (fourth edition, Biringen, 2008)^12^. All four raters were trained and certified by Zeynep Biringen and blinded to the dyads intervention group. They achieved an interrater reliability for the global ratings of ICC = 0.942–0.996 and for the total ratings of ICC = 0.944–0.966 (average-measure intra-class correlations), indicating an excellent agreement (Cicchetti, 1994).

The EAS incorporates elements of system theory and of the concept of EA originally developed by Emde (1980; Emde and Easterbrooks, 1985) for the psychotherapeutic setting. Emde (1980) described EA as followed: “EA refers to an individual’s emotional responsiveness and ‘attunement’ to another’s needs and goals; key is the acceptance of a wide range of emotions rather than responsiveness solely to distress” (p. 80). Furthermore, EA, as described by Biringen (2000) is a way of assessing the relationship between a caregiver and child, be it mother, father, or another caregiver. The EAS allow for a detailed look at caregiver (parent)-child interactions by rating the dyad on six dimensions. Parental *sensitivity*, as known from attachment research, is a construct that takes into account qualitative factors such as affect, timing, flexibility, acceptance, conflict negotiation, and the parent’s awareness of their child’s cues as well as appropriate responsiveness. Parental *structuring* refers to the parent’s ability to structure or scaffold the child’s environment and play. Parental *non-intrusiveness* involves the degree to which the parent can be available without interfering with the child’s autonomy and space. Parental *non-hostility* refers to parental behavior that is free of impatience, harshness or malice. Child *responsiveness* to the caregiver refers to the child’s ability to explore on its own as well as respond to the caregiver in an affectively positive way. This is reflected in the child’s interest, eagerness, and pleasure following a parental bid for interaction. Child’s *involvement* of the caregiver refers to the child’s ability to attend to and engage the caregiver in interactions and invite the caregiver into play. Each dimension is rated globally as well as with the help of seven subscales on a scale of one to seven (first two scales) or one to three with higher scores indicating a higher EA.

The EAS were applied in order to obtain a more complex and a more emotionally than behaviorally oriented impression of EA than it is often the case in classical attachment research paradigms (Biringen et al., 2014; Lebiger-Vogel, 2016). In contrast to approaches using counts of discrete behaviors EA is a global and holistic judgment by which the observer uses contextual cues and clinical judgment to infer the appropriateness of behavior. The EAS are an observational instrument with a dyadic focus, meaning high scores on the adult scales can only be achieved if the child is appropriately responsive and involving. Thus EA is a relational construct involving emotional expression and responsiveness during dyadic interactions, and emphasizes the bidirectional quality of the emotional dialog between parent and child in a relationship. Correspondingly, EA refers not only to the parent’s emotional signals, but also to the emotional signals sent by the child, and the parent’s ability to interpret and understand the child’s emotional experience (Biringen, 2000; Easterbrooks and Biringen, 2000). An emotionally available mother uses a sensitive, structuring, non-intrusive, and non-hostile style of caregiving that facilitates the child’s ability to successfully regulate emotion and behavior. This in turn enables the child to reciprocate in a responsive and involving manner towards the mother (Biringen, 2000; Easterbrooks and Biringen, 2000).

The scales show acceptable reliability estimates and their validity has been reported in several studies (e.g., Aviezer et al., 1999; Easterbrooks and Biringen, 2000, 2005; Bornstein et al., 2006). In addition, they have been used to examine caregiver-child relationships across a broad spectrum of developmental ages, starting with early childhood until the beginning of adolescence (e.g., Zimmerman and Fassler, 2003; de Falco et al., 2009; Vliegen et al., 2009; Biringen et al., 2010; Easterbrooks et al., 2012).

### Scalp Hair Collection and Cortisol Measurement

In the current study hair samples were collected during home visits approximately 7–10 days (mean = 8.17 days; *SD* = 3.40) before kindergarten entry (HC1) as well as approximately 3 months (mean = 3.25 months; *SD* = 0.32) afterwards (HC2)^13^. Assessments were conducted by the first author or trained graduate students. At each time of measurement a scalp hair strand was collected from the posterior vertex position by cutting the hair as close to the scalp as possible. These were placed onto aluminum foil, stored in a dry, dark place at room temperature, until sent for analysis to the lab of Prof. Dr. Kirschbaum (Faculty of Psychology, Department of Biopsychology, University of Dresden, Germany). Hair analysis was conducted for both times of measurements using one 3-cm long segment. Based on an average monthly hair growth of approximately 1 cm, each segment represented the averaged cortisol cumulated over an approximate timespan of 3 months. Thus the two segments analyzed for the two times of measurement reflected the children’s mean cumulated HCC during the first 3 months of kindergarten attendance (HC2) and 3 months prior to kindergarten entry (HC1). At each time of measurement parents received a questionnaire with questions about the child’s age, hair characteristics (hair washing frequency, hair color, waves/curves, hair treatment/dyeing), medication and disease (use of corticosteroids and other medication during the last 3 months, chronic disease) as well as previous changes and challenges in the child’s life during the last 3 months. Three dyads with children who reported to use medications thought to interfere with cortisol assays (e.g., inhaler for asthma, allergy medication) had been excluded from the study. None of the children suffered from any chronic disease or had any hair treatment/dyeing.

Over the past years supportive evidence has accumulated regarding several fundamental characteristics of HCC, including its validity as an index of long-term systemic cortisol levels both in animals (e.g., Fairbanks et al., 2011) and in human participants (e.g., Manenschijn et al., 2011), its reliability across repeated assessments (Stalder et al., 2012b) and its relative robustness to a range of potential confounding influences (for a review see Stalder and Kirschbaum, 2012).

### Data Analysis

Differences in the cumulative HCC before and after kindergarten entry were calculated and analyzed with the help of a one-sample *t*-test. Additionally, the relationships between different EA scales and the difference in HCC before and after kindergarten entry were analyzed with the help of Spearman correlation coefficients. An ANCOVA for repeated measures^14^ was tested with one within-subject factor “change in cortisol level” (HC1 vs. HC2) and six between-subject factors, one for each dimension. For the between-subject factors dyads were divided in two groups for each EA dimension, one consisting of dyads with scores of 5–7 and one consisting of dyads scoring under 5 (“sensitivity”: low vs. high; “structuring”: low vs. high; “non-intrusiveness”: low vs. high; “non-hostility”: low vs. high; “child responsiveness”: low vs. high; “child involvement”: low vs. high). The cut-off score of 5 was chosen because according to Biringen (2008) scores of 5 and higher indicate emotional available interactions while scores lower than 5 indicate problematic emotional available and emotional unavailable interactions. Two potential confounding variables, children’s age at kindergarten entry and mother’s duration of school education (in years)^15^, were additionally included as covariates^16^.

## Results

### Change in HCC

**FIGURE 1:**
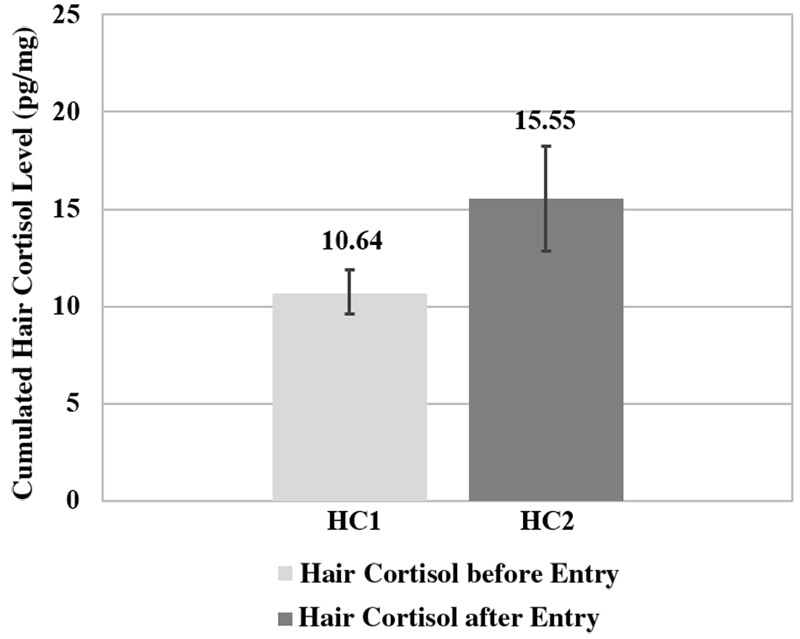
**Mean cumulated hair cortisol levels during the 3 months before (H1) and 3 months after kindergarten entry (H2) of *N* = 24 children**.

The mean cumulated value of HCC reflecting the 3 months prior to kindergarten entry was 10.64 (*SD* = 6.46) and the one reflecting the 3 months after kindergarten entry was 15.55 (*SD* = 13.14; see **Figure 1**). This resulted in a mean difference between both times of measurement of 4.91 (*SD* = 12.28) indicating a rise of cortisol after kindergarten entry compared to the time prior. Additionally, the one-sample *t*-test revealed a significant difference in cortisol between the two times of measurement [*t*(23) = 8.063, *p* < 0.000]. Testing potential differences in the rise of cortisol between those children who had visited another child care institution before kindergarten entry (*n* = 6) and those who had been cared for at home using a Mann–Whitney-*U*-test (due to violation of variance homogeneity) revealed no significant differences (*z* = -0.467; *p* = 0.673). No significant difference in the rise of cortisol was found between boys and girls (*z* = 0.468; *p* = 0.666), again using the Mann–Whitney-*U*-test (due to violation of variance homogeneity).

### Emotional Availability

The mean values concerning the different EA dimensions show varying scores. Furthermore, the amount of dyads who were allocated to the groups with high scores (>4.5) on the different dimensions was higher than the amount of those who were rated lower (<5; see **Table 2**).

**Table 2:** Mean scores of the Emotional Availability Scales (EAS) and standard deviations (*SD*) of *N* = 24 mother–child dyads as well as subsample sizes of the generated groups for ANCOVA.

EA dimensions	Mean	*SD*	Minimum	Maximum	*n* with high scores (>4.5)	*n* with low scores (<5)
Mother’s sensitivity	4.75	1.15	2.50	6.50	*n* = 15	*n* = 9
Mother’s structuring	4.58	1.40	2.00	7.00	*n* = 12	*n* = 12
Mother’s non-intrusiveness	4.58	1.32	2.00	7.00	*n* = 14	*n* = 10
Mother’s non-hostility	5.54	1.08	2.50	7.00	*n* = 21	*n* = 3
Child responsiveness	4.92	1.10	2.00	6.50	*n* = 16	*n* = 8
Child involvement	4.63	1.18	2.50	6.50	*n* = 14	*n* = 10

### Change in Cortisol and the Emotional Availability

Testing the relationship between the change in mean cortisol level and the different EA dimensions revealed a significant negative correlation between the rise in cortisol after kindergarten entry and mother’s sensitivity (*r* = -0.45, *p* = 0.028). Four of the dimensions correlated marginally with the change in cortisol (structuring: *r* = -0.388, *p* = 0.061; non-intrusiveness: *r* = -0.366, *p* = 0.079; child responsiveness: *r* = -0.355, *p* = 0.089; child involvement: *r* = -0.366, *p* = 0.078). However, the correlation coefficient between the change in mean cortisol level and the dimension mother’s non-hostility turned out to be not significant (*r* = -0.334, *p* = 0.110).

**FIGURE 2:**
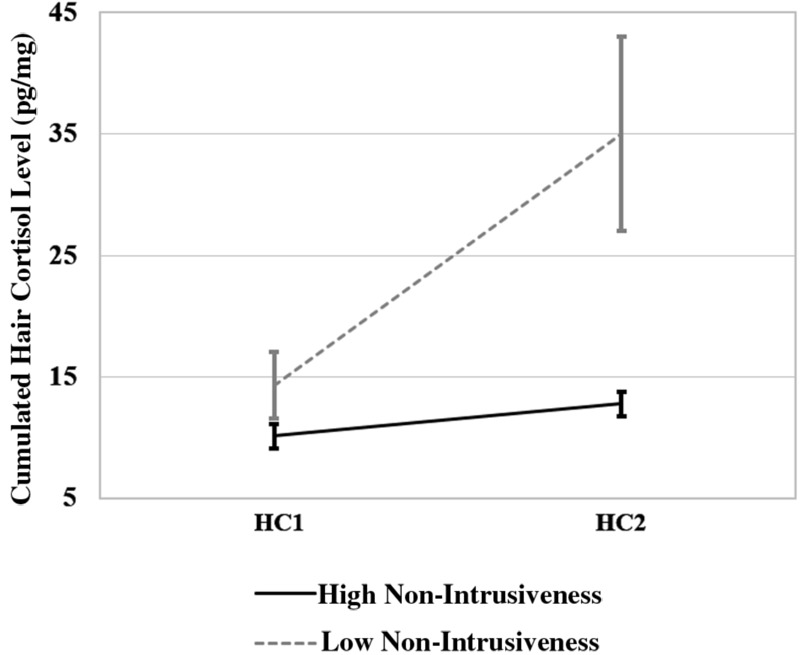
**Interaction between “change in cortisol level” (HC1 vs. HC2) and “non-intrusiveness” (high vs. low non-intrusiveness)**.

**FIGURE 3:**
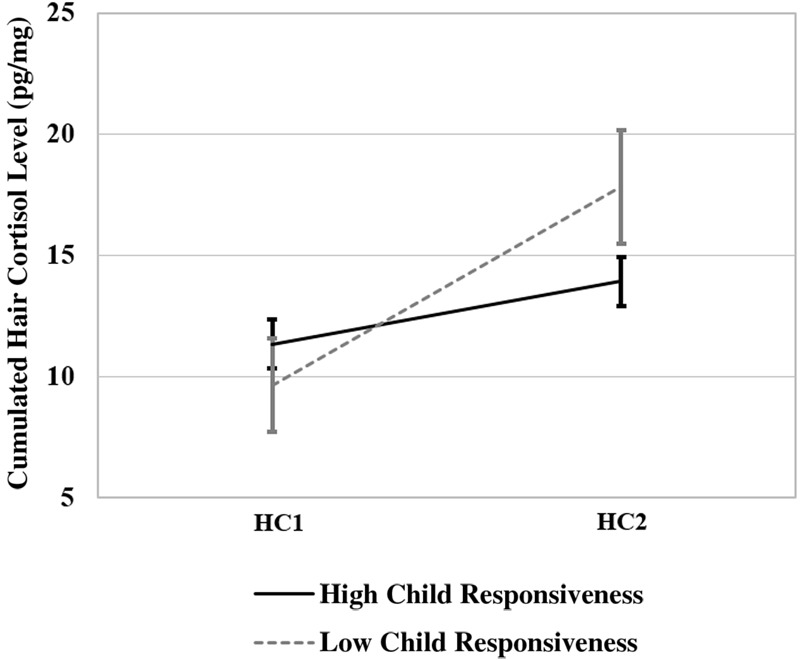
**Interaction between “change in cortisol level” (HC1 vs. HC2) and “child responsiveness” (high vs. low responsiveness)**.

Additionally, the effects of the EA dimensions were tested using a repeated measures ANCOVA with one within-subject factor “change in cortisol level” (HC1, HC2) and six between-subject factors: 1. “sensitivity” (low vs. high), 2. “structuring” (low vs. high); 3. “non-intrusiveness” (low vs. high); 4. “non-hostility” (low vs. high); 5. “child responsiveness” (low vs. high), and 6. “child involvement” (low vs. high). Besides the significant main effects for “change in cortisol level” [*F*(1,10) = 13.010, *p* < 0.007], and “child responsiveness” [*F*(1,10) = 14.837, *p* = 0.003] the ANCOVA revealed a significant interaction between “change in cortisol level” and “non-intrusiveness” [*F*(1,10) = 9.608, *p* = 0.011); see **Figure 2**] and “change in cortisol level” and “child responsiveness” [*F*(1,10) = 36.324, *p* = 0.000; see **Figure 3**]. All other main effects and interactions were not significant.

Furthermore, both children’s age at kindergarten entry and mother’s duration of school education in years had no significant effects.

## Discussion

### The Rise in Cortisol after Kindergarten Entry

As expected, statistical analysis demonstrated a significant rise in cortisol after kindergarten entry, indicating children show higher cumulated physiological stress levels during the 3 months after kindergarten entry compared to the months prior. This result is in line with previous studies that have demonstrated elevations in cortisol after transition to child care (e.g., Ahnert et al., 2004; see review Vermeer and van IJzendoorn, 2006; Watamura et al., 2009) or school (Groeneveld et al., 2013). Due to the small sample size these differences might be even more pronounced in a bigger sample. Apart from this, expectation of kindergarten entry might have already led to elevations in HCC prior to kindergarten entry. Descriptive reports by the FIRST STEPS project staff indicate that the mother–child dyads are already preoccupied with the transition to kindergarten and associated changes weeks before the actual beginning of kindergarten entry (Rickmeyer, 2016; Rickmeyer et al., in press). Therefore, in future research it would be interesting to additionally assess HCC long before kindergarten or child care entry in order to compare it with measurements after entry. This might lead to even higher differences in cortisol levels. Statistical analyses demonstrated no effects of previous child care experiences on the rise in cortisol. However, this result might stem from the small subsample size of only *n* = 6 children who had visited another child care institution before kindergarten entry. It could be speculated that previous child care experiences facilitate the transition to kindergarten, leading to a smaller rise of cortisol after entry. This effect could be particularly pronounced in children with an immigrant background for whom the transition from home to out-of-home child care may be particularly challenging and stressful due to parents’ migration-related losses (Schaich, 2012; Rickmeyer, 2016; Rickmeyer et al., in press). On the other hand, previous research has shown that even after the transition to school, when the children have normally already experienced out-of-home care for a long time, children show elevations of cortisol (Groeneveld et al., 2013). Taking into account the assumed buffering role of attachment to teachers in association with elevations of cortisol due to child care (Badanes et al., 2012), these elevations could be explained by the fact that every transition to a new form of out-of-home child care (e.g., preschool or primary school) is normally associated with a change of teachers. Thus in every new form of out-of-home care children need time to build relationships to their new teachers who may function as new attachment figures.

Furthermore, it could be speculated that particularly those children who start child care earlier in life cope with stress induced by kindergarten entry less effectively due to a higher cortisol reactivity caused by early child care experiences. However, further research is needed in order to investigate the exact effects of early out-of-home child care experiences. Another variable that might affect the increase of elevations due to child care could be children’s age. For example, a study by Watamura et al. (2003) demonstrated differences in the rise in cortisol across the day at child care between *n* = 35 toddlers (mean age = 29.7 months) and *n* = 20 infants (mean age = 10.8 months) with more toddlers showing increasing cortisol levels across the day than infants. Thus future research could aim at investigating infant’s HCC before and after entry to “Kinderkrippe” or other earlier out-of-home child care in order to test whether a rise of cumulated cortisol would be found in younger children, too. In addition, it could be assumed that the rise in cortisol found in our sample could be explained by variations of HCC due to age, rather than an increase in stress due to kindergarten entry. However, studies on non-human primates (e.g., Laudenslager et al., 2012; Meyer and Novak, 2012) as well as humans indicate that HCC does not increase with age (Dettenborn et al., 2012). For example, the study by Dettenborn et al. (2012) found HCC to be elevated both in younger children and older adults. Furthermore, the children’s age at hair cortisol assessments varied in our sample.

### Emotional Availability of the Sample

In comparison to previously reported EA dimension mean scores as well as taking into account the assumption that a dimension score of below five indicates a mother–child interaction in a problematic zone, all dimension scores of our sample, besides the mean score of mother’s non-hostility, indicate a decreased, but not alarming EA within the mother–child relationships. Compared to the sample of refugees and asylum-seeking mothers and children who were included in the study by van Ee et al. (2012) our sample demonstrates only slightly higher mean scores on all EA dimensions^17^. This comparison could be limited by the fact that compared to the children included in our study the children in the study by van Ee et al. (2012) were on average younger (mean age = 26.6 months, range = 16–46 months). Furthermore, it could be speculated that our sample is less traumatized than the sample by van Ee et al. (2012) because our study included mainly women who immigrated due to marriage, family reunification, and economic reasons rather than refugees and asylum-seeking mothers. However, the only slightly higher mean scores of our sample in comparison to the sample by van Ee et al. (2012) underline the conclusion that the scores of our sample are relatively low. Another study on US subcultural comparisons by Derscheid (2012) compared Hispanic (*n* = 20), African American (*n* = 20), and Caucasian dyads (*n* = 10) at children’s age of 2–4 years, matched on different demographic variables (maternal age, education, and child gender). Thereby no significant differences in EA dimension mean scores were found between Hispanic and African-American race (ethnic groups) and Caucasian dyads (comparison group), suggesting similarity across these US subcultures. However, the sample was relatively small. In comparison to the *N =* 40 dyads investigated by Derscheid (2012) our sample shows lower mean scores on all EA dimensions. However, in comparison to high-risk dyads (*N* = 34) of mothers with substance-use difficulties and their children (age of 12–40 months) investigated by Espinet et al. (2013) our sample demonstrates higher scores on all EA dimensions: mother’s sensitivity (FIRST STEPS: 4.75 vs. Espinet et al., 2013: 3.82), structuring (FIRST STEPS: 4.58 vs. Espinet et al., 2013: 3.88), non-intrusiveness (FIRST STEPS: 4.58 vs. Espinet et al., 2013: 3.59), non-hostility (FIRST STEPS: 5.54 vs. Espinet et al., 2013: 3.94), child responsiveness (FIRST STEPS: 4.92 vs. Espinet et al., 2013: 3.59), and child involvement (FIRST STEPS: 4.63 vs. Espinet et al., 2013: 3.38). To conclude, our sample demonstrates decreased, but not alarming EA mean scores indicating complicated emotional available mother–child relationships and a need for intervention.

### Negative Relationship between the Rise in Cortisol and the Emotional Availability

The significant negative correlations between the rise in HCC and five of the EA dimensions indicate that the EA played a role in the rise of cumulated HCC after kindergarten entry. However, correlations should be treated with caution and do not represent causal effects. Variance analyses demonstrated that mother’s intrusiveness as well as child responsiveness have significant effects on the rise in cortisol, with lower cortisol increases in responsive children and children who have experienced less intrusive mother–child relationships. Taking into account the small sample size, this is a particularly interesting finding. It is in line with the previous assumption that children who have experienced more emotional available mother–child relationships may be better equipped to deal with challenges induced by child care entry (Ahnert et al., 2004; Rickmeyer, 2016). In addition, it is consistent with studies that indicate that more emotional available relationships with the primary care-giver help to better regulate emotions and stress responses in general (Little and Carter, 2005; Kertes et al., 2009). Because of the above mentioned risk of separation conflicts due to parents’ migration-related losses it is a particularly interesting result that mothers’ intrusiveness seems to influence the rise in cortisol after kindergarten entry. It could be speculated that mothers who have experienced losses due to migration and thus are particularly vulnerable to separations tend to deny their children’s autonomy and are more likely to be overprotecting and thus more intrusive. This in return could complicate children’s transition to kindergarten, an important step towards more autonomy (Rickmeyer, 2016; Rickmeyer et al., in press). The influence of child responsiveness on the rise of cortisol after kindergarten entry could be explained by the assumption that children who are more responsive due to their previous experiences in the relationship to their primary caregiver can get in contact with kindergarten teachers more easily (Biringen et al., 2014). Thus they might be better equipped to build up secure relationships to their kindergarten teachers than children who show less responsiveness. This in return may help them to deal more effectively with the challenges induced by child care entry, compared to less responsive children, leading do a smaller increase in cortisol after kindergarten entry.

### Limitations and Future Research

Several additional limitations should be noted. First, as mentioned above, in view of statistical analyses a larger sample size is considered desirable. Apart from this, other variables that seem to influence the increase of cortisol during child care such as caregiving quality (Tout et al., 1998; Dettling et al., 2000; Sims et al., 2006; Watamura et al., 2009) and attachment to teachers/child care providers (Badanes et al., 2012) have not been assessed and therefore could not be controlled for in this study. Given the applicability of the EAS in child care contexts it would be of particular interest to assess the emotional quality of children’s relationship to kindergarten teachers with the help of the EAS. Previous research indicates that numerous dimensions of EA have been predictive of attachment to teachers/child care providers, suggesting the appropriateness of dyadic EA evaluation in multiple caregiver contexts (see review by Biringen et al., 2014). Another potential confounding variable, that has not been assessed, is mother’s traumatization. Given that lower mother-child EA has been found in circumstances where the mother is traumatized by war (e.g., van Ee et al., 2012), mother’s potential traumatization or posttraumatic stress symptoms should be assessed and tested in future research.

Additionally, due to the small sample size the effects of other potentially confounding variables (e.g., exact length of the adaptation phase, previous changes and challenges in life during the last 3 months, daily hours spent at kindergarten, type of transition model, previous child care experiences, child rank and mother’s length of stay in Germany, etc.) could not be tested and should be investigated in a larger sample, too. The same applies for children’s hair characteristics (hair color, waves/curls) and hair washing frequency. Both might have effects on children’s HCC. However, research has reported no influence of natural hair color (Raul et al., 2004; Sauvé et al., 2007; Kirschbaum et al., 2009; Manenschijn et al., 2011) and hair characteristics, such as waves or curls, (Dettenborn et al., 2012) on HCC. Furthermore, the frequency of hair washing is likely to have only a small effect: Research has not revealed an effect of the frequency of hair washing on HCC in proximal hair segments (Manenschijn et al., 2011; Dettenborn et al., 2012; Stalder et al., 2012a), although there was some indication that more frequent hair washing may be related to reduced HCC in more distal hair segments (Dettenborn et al., 2012). However, the latter does not apply in the current study. Furthermore, taking into account inconsistent results on a potential relationship between ethnicity or country of origin and HCC (e.g., O’Brien et al., 2013; Wosu et al., 2015), potential effects of these variables should be considered and tested in a larger sample, too.

Another methodological limitation is the validation of the used methods. The EAS have been applied in at least 22 countries all over the world and show adequate reliability and validity in each of these cultures (see review by Biringen et al., 2014). However, only a few studies have actually investigated cross-cultural comparisons to understand levels of EA in different societies. To date the only published study on cross-national comparisons, using the EAS, is a study by Bornstein et al. (2008) who investigated *N* = 220 dyads from Italy, Argentina, and the US. Their results demonstrated that Italian mothers showed higher scores in sensitivity and structuring and their children were more responsive and involving than in dyads from Argentina and the US. As mentioned above, another study on US subcultural comparisons by Derscheid (2012) indicated no significant differences in EA between Hispanic, African American, and Caucasian dyads. However, the sample size might have been too small to detect relevant significant differences. Thus further studies with higher sample sizes would be desirable.

Additionally, there was no comparison group of toddlers who did not enter child care and who had not taken part in the intervention study (FIRST STEPS) or toddlers without an immigrant background. Therefore we firstly do not know if HCC or EA, or both, would have changed over this age period in the absence of kindergarten. Secondly, we do not know whether children without an immigrant background as well as immigrant children, who did not take part in our intervention study, would also show a rise in the mean cumulated HCC after kindergarten entry as well as associations between a potential rise in cortisol and EA dimensions (mother’s intrusiveness and child responsiveness). These questions could be examined in future investigations.

Another critical point is that due to the FIRST STEPS study design the time span between EAS assessment and kindergarten entry varied individually. While *n* = 5 dyads were video-taped after kindergarten entry, the remaining *n* = 19 dyads were recorded on video prior to entry. Although research indicates a stability of EA over several months (Biringen, 2000), the varying time between EA assessment and kindergarten entry might have affected the found associations between the rise in HCC and the two EA dimensions (mother’s intrusiveness and child responsiveness).

In the FIRST STEPS study children’s HCC is additionally assessed 1 year after kindergarten entry in order to investigate whether elevations in cortisol decrease again or remain increased after a longer time spent in kindergarten. In line with the assumptions by Badanes et al. (2012), it could be speculated that with increasing months spent at kindergarten the effects of maternal EA on potential elevations of cortisol in association with child care decrease and that the influence of the attachment to teachers increases. Nevertheless it could be interesting to investigate whether maternal EA still has an effect on children’s HCC 1 year after kindergarten entry. However, to date not enough data of this time of measurement is available, yet.

## Conclusion and Implications

This study is unique for its thorough observation of parent-child interactions among children with an immigrant background in association with potential elevations in cortisol due to kindergarten entry. Furthermore, to our knowledge elevations in cortisol due to child care entry have not been studied with the help of hair cortisol assessment, yet. Our results indicate, as expected, that children demonstrate higher levels of cumulated cortisol during the challenging time after kindergarten entry compared to the months prior. Thereby two dimensions of EA, mother’s non-intrusiveness and children’s responsiveness, seem to play an important buffering role. Thus taking into account potential negative effects of early stress and associated elevations in cortisol, our results demonstrate how important an empathetic support of children during child care entry (such as kindergarten entry) is. Based on our results it could be assumed that supporting especially both mother’s non-intrusiveness and children’s responsiveness in dyads with an immigrant background could help to decrease potential elevations of cortisol in association with child care entry, thus reducing stress in this critical phase early in life. Furthermore, interventions promoting intercultural parent work and the EA of early child care and kindergarten teachers could facilitate the empathetic support of these children during the transition to child care, too (Shivers, 2008). Thereby trainings that sensitize early child care and kindergarten teachers to the specific challenges of families with an immigrant background in association with child care entry (e.g., due to migration-related losses) could be of particular relevance (Schaich, 2012). Furthermore, research suggests that promoting secure attachment to professional caregivers can be of particular importance for children living in difficult life circumstances and growing up with less sensitive parents in order to prevent negative developmental outcomes (Hauser et al., 2009).

In addition, research among immigrant families is still scarce, but tremendously needed. Not only is there a global increase in immigrants in Germany and other European countries, but children with an immigrant background are still disadvantage in our educational system and are more likely to grow up in high-risk environments (Autorengruppe Bildungsberichterstattung, 2016). The integration of these children is one of the most important challenges for European societies (and other western countries). Supporting their integration is important in order to reduce the risk that these children feel excluded and not wanted by the majority society as well as to prevent or at least reduce social divides, tensions, social withdrawal into subcultural milieus as well as the risk of radicalization (Meurs, 2016).

It is important to note, that on the basis of our results, we cannot conclude that stress due to the transition to kindergarten has either negative or positive effects. The present study only demonstrates that children with an immigrant background showed elevated cumulated cortisol levels in response to kindergarten entry and that this effect seems to be particularly pronounce in less responsive children and children with more intrusive mothers. However, taking into account potential negative effects of cortisol elevations early in life, supporting mother’s non-intrusiveness and children’s responsiveness seems to be a promising approach in order to reduce potential elevations of cortisol in association with kindergarten entry and thus promoting early child development. Previous studies have shown that the EA of a dyad is relatively stable across time, but that it is sensitive to change through interventions supporting emotional available caregiver-child relationships at home as well as in professional caregiving settings (Biringen et al., 2010, 2012, 2014; Baker et al., 2015).

## Author Contributions

All authors made substantial contributions to conception and design, acquisition of data as well as interpretation of data. CR drafted the article and analyzed the data, while ML-B and JL-V revised the article critically for important intellectual content. Additionally, JL-V supported the ratings of the videos used for data analysis. All authors gave final approval of the version to be submitted and agree to be accountable for all aspects of the work in ensuring that questions related to the accuracy or integrity of any part of the work are appropriately investigated and resolved.

## Conflict of Interest Statement

The authors declare that the research was conducted in the absence of any commercial or financial relationships that could be construed as a potential conflict of interest.

## Footnotes

1 In Germany kindergarten starts around the age of three until the children are 6 years old and enter primary school. Due to the introduction of the legal right to child day care for children between one and three in 2013 an increasing amount of children visits the “Kinderkrippe" (crèche, day nursery) before entering kindergarten. However, in 2015 there was still a high amount of children (78% of children with an immigrant background and 62% of children without an immigrant background) that stayed at home until the transition to kindergarten (Autorengruppe Bildungsberichterstattung, 2016).

2 This typical pattern develops early in life. However, it seems to be observed more reliable in children that are older than 3 years than in younger children (Bruce et al., 2002; Watamura et al., 2003).

3 For example, in two studies conducted in Russian and Romanian children’s homes children showed blunted rhythms of diurnal cortisol production with relatively low cortisol levels in the morning and only a small or no decline of cortisol levels across the day (Carlson and Earls, 1997; Gunnar, 2000).

4 The NICHD Study of Early Child Care and Youth Development (SECCYD) is a comprehensive longitudinal study from birth until the end of sixth grade. It has been initiated by the NICHD (National Institute of Child Health and Human Development) to answer questions about the relationships between child care experiences, child care characteristics and children’s developmental outcomes. It started in 1991 and has included *N* = 1.364 children.

5 Pre-kindergarten is a classroom-based preschool program for children at or below the age of five in the US, Canada, and Turkey.

6 Seventy-five percent of the children included in the current study did not attend any other child care institution before kindergarten. Although in Germany the total amount of children who visit child care before kindergarten has increased due to changes in the child care system (see above), children with an immigrant background are still less likely to attend out-of-home child care before kindergarten compared to children without an immigrant background (Schober and Stahl, 2014; Autorengruppe Bildungsberichterstattung, 2016).

7 In this study hair samples reflecting the first 2 months after as well as 2 months before school entry were analyzed. The results demonstrated that the beginning of elementary school is accompanied by increased cumulated cortisol levels, too. This increase was particularly pronounced in fearful children.

8 IDEA = Individual Development and Adaptive Education of Children at Risk; http://www.idea-frankfurt.eu/; IDeA represents a research cooperation of the Goethe University Frankfurt/Main, the German Institute for International Educational Research (DIPF) and the SFI.

9 The project has been inspired by the *First Steps* project of Patrick Meurs who began with the project in Belgium in 2000 (Meurs et al., 2006; Meurs and Jullian, 2016).

10 The FIRST STEPS approach is curriculum-based and includes psychoanalytic case-supervision, practice reflection as well as supervision with child and adolescent psychoanalysts for the project staff (Burkhardt-Mußmann, 2015).

11 “Hard-to-reach” refers to the difficulty to reach or involve certain sub-groups of population in research or public health.

12 In the FIRST STEPS study the EAS are additionally rated after videotaping mother–child interaction during natural feeding and care-taking situations for 30 minutes during the intervention (with 2.5–5.5 months, 13–15 months, 24–27 months; for a description of the application of the EAS see Lebiger-Vogel, 2016). Another advantage of the EAS is that they are sensitive to change related to a program of intervention (Biringen et al., 2014).

13 Due to the families‘ life circumstances it was not possible to always assess the hair samples exactly 7 days before and 3 months after kindergarten entry. Within the FIRST STEPS study hair cortisol samples are additionally collected 1 year after kindergarten entry (HC3). However, to date hair cortisol data at HC3 of only *n* = 7 children are available.

14 Requirements for both repeated measures ANCOVA and *t*-test were met.

15 Mother’s duration of school education was used as an indicator of mother’s SES (Sirin, 2005), because SES has not been assessed.

16 Due to the small sample size further confounding variables were not included in the model.

17 Mother’s sensitivity (FIRST STEPS: 4.75 vs. van Ee et al., 2012: 4.27), structuring (FIRST STEPS: 4.58 vs. van Ee et al., 2012: 4.31), non-intrusiveness (FIRST STEPS: 4.58 vs. van Ee et al., 2012: 4.37), non-hostility (FIRST STEPS: 5.54 vs. van Ee et al., 2012: 5.00), child responsiveness (FIRST STEPS: 4.92 vs. van Ee et al., 2012: 4.27), and child involvement (FIRST STEPS: 4.63 vs. van Ee et al., 2012: 4.06).
